# Misconceptions and Insights about Flame Tests

**DOI:** 10.1021/acs.jchemed.5c00142

**Published:** 2025-09-09

**Authors:** Michael A. Duncan

**Affiliations:** Department of Chemistry, 1355 University of Georgia, Athens, Georgia 30602, United States

**Keywords:** First-year/general, Laboratory instruction, Misconceptions/discrepant events, Atomic spectroscopy

## Abstract

Flame tests, in which aqueous metal salt solutions on a wire inserted into a flame produce colored emissions, are one of the simplest demonstrations used in chemistry. Unfortunately, the phenomena giving rise to the colored emissions is poorly understood by many and often described incorrectly in textbooks and published articles. In the present commentary, the details of flame tests are discussed in the context of well-established atomic spectroscopy. It is shown that the emission does not come from the ions which are present in solution but rather usually comes from the excited states of the corresponding neutral atoms. Details of the mechanism, involving ion desolvation and gas-phase electron transfer, are discussed.

## Graphical abstract

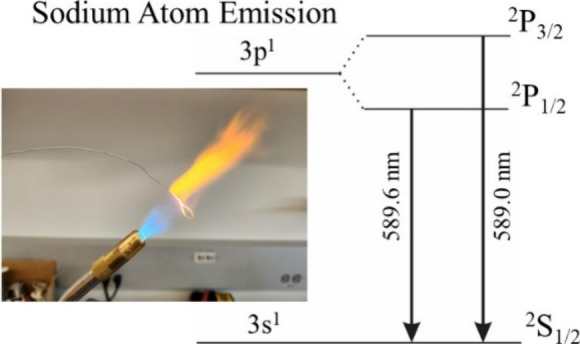

Flame tests have been used for elemental analysis since the earliest days of chemistry, when the elements were first isolated and identified. For example, Humphry Davy used flame tests to characterize the alkali metals when he first isolated sodium and potassium in 1807.^1^ Gustav Kirchoff and Robert Bunsen used Bunsen’s newly developed burner to obtain the first flame emission spectra and discovered the new elements cesium and rubidium in 1859.^2^ In modern general chemistry classes, flame tests are employed extensively to introduce atomic theory and qualitative elemental analysis. Because the equipment involved is simple (Bunsen burner, salt solution, wire), this is one of the most widely employed demonstrations/experiments in high school or freshman chemistry classes. Flame tests are described in many General Chemistry textbooks,^3–12^ on Web sites,^13–16^ and also on streaming sites such as YouTube.^17–19^ Several articles in the chemical education literature^20–23^ and one doctoral dissertation^24^ have discussed these tests and their interpretation. Unfortunately, while the qualitative aspects of these tests are generally accepted, i.e., the colors resulting for different elements, the interpretation of the process giving rise to colored emission varies in different sources. Specifically, the chemical species giving rise to emission in flame tests are sometimes ascribed to the metal *ions* which exist in solution, whereas other sources assign the emission to the *neutral* metal atoms. The electronic structure of neutral metal atoms versus the corresponding ions are very different, and so this contradiction can be confusing to students. Many texts just do not make it clear what the specific source of the emission is. This commentary is designed to elucidate the origin of colored emissions seen in common flame tests.

The specific atomic-level details of flame colors are not generally well understood. On examination of selected General Chemistry textbooks, several of these made no mention of the specific source of the emission other than the elements present.^3–5^ Some texts do not discuss flame tests but instead mention fireworks with atomic emission as the source of the colors.^6^ Other sources focus on the lack of understanding of how atoms are excited in the flame and whether or not energy is gained or lost in the emission process.^21,22,24^ Some textbooks and research articles clearly attributed the emission to the *cations* present in solution,^7–10,20,23^ whereas others assigned the emission to the *neutral atoms*.^11,12^ Wikipedia assigns the emission to ions, as shown in the following quote:^13^

“In flame tests, ions are excited thermally. These excited states then relax to the ground state with emission of a photon.”

ChatGPT is not regarded as a reliable scientific source, and its information is changing constantly. Nevertheless, this source is often consulted by students and may be a source of their confusion. A simple question such as that below produces the following result:^25^

Question: Write a paragraph about the flame test in chemistry?

Result: The flame test is a qualitative analysis technique used in chemistry to identify the presence of certain metal ions based on the characteristic color they emit when heated in a flame. When a metal salt is introduced to a flame, the heat excites the electrons in the metal ions, causing them to jump to higher energy levels. As the electrons return to their original energy levels, they release energy in the form of visible light. Different metal ions emit different colors—for example, sodium produces a bright yellow flame, copper yields a green or blue flame, and potassium shows a pale violet flame. This simple yet effective method helps chemists quickly identify metal elements in a sample.

This is assigning the emission to the ions. If the question is posed in a different way, mentioning the NIST Atomic Spectra Database^26^ (vida infra) as a resource, other responses indicate that the emission comes from neutral atoms. Other online sources such as the Chemistry LibreTexts^14^ or the PhysicsOpenLab^15^ likewise attribute the emission to the neutral atoms. The conclusions of these various sources are therefore inconsistent with each other and require clarification.

Atomic spectroscopy is a mature field, although its details are not often covered in General Chemistry. The same atomic transitions that appear in flames are used in countless applications, from fireworks,^27^ to atomic absorption spectroscopy in analytical chemistry,^28^ to astrophysics and the classification of stars,^29^ to optical physics and laser cooling.^30^ The emission spectra produced in these various experiments are analyzed with spectrometers, providing the specific wavelengths of light produced. Because of this, the atomic transitions for most of the elements in different charge states have been known for over a hundred years. These transitions are documented in standard databases, such as the NIST Atomic Spectra Database,^26^ which is the modern-day repository for data that was meticulously organized over many years by Dr. Charlotte E. Moore,^31^ among others. Instead of just assigning qualitative colors to flame emissions, it is therefore also possible to measure the spectra of these flames with spectrometers, and to determine the exact wavelengths of light produced. With such wavelengths, the transitions giving rise to the emissions for each element can be compared with those in the NIST database and the specific charge state and electronic transition(s) of the metal can be identified. The PhysicsOpenLab Web site^15^ shows how this is done, and the Knowledge. Carolina site^16^ provides examples of the spectra for selected metals. Table 1 lists some of the prominent emission colors for selected elements from flame tests and the assignments made in this way.

**Table 1:** Selected Atomic Emission Transitions for Representative Metal Flames

Metal	Color	λ (nm)	Assignment
lithium	red	670.8	Li (2p → 2s; ^2^P_1/2,3/2_ → ^2^S)
sodium	yellow	589.0	Na (3p → 3s; ^2^P_3/2_ → ^2^S)
		589.6	Na (3p → 3s; ^2^P_1/2_ → ^2^S)
potassium	lilac	766.5	K (4p → 4s; ^2^P_3/2_ → ^2^S)
		769.9	K (4p → 4s; ^2^P_1/2_ → ^2^S)
calcium	orange	643.9	Ca (3p^6^3d^4^4p^1^ → 3p^6^3d^4^4s^1^; ^3^F_4_ → ^3^D_3_)
		646.3	Ca (3p^6^3d^4^4p^1^ → 3p^6^3d^4^4s^1^; ^3^F_3_ → ^3^D_2_)
		649.4	Ca (3p^6^3d^4^4p^1^ → 3p^6^3d^4^4s^1^; ^3^F_2_ → ^3^D_1_)
strontium	crimson	640.8	Sr (4d^1^5p^1^ → 4d^1^5s^1^; ^3^F_4_ → ^3^D_3_)
		650.4	Sr (4d^1^5p^1^ → 4d^1^5s^1^; ^3^F_3_→ ^3^D_2_)
		661.7	Sr (4d^1^5p^1^ → 4d^1^5s^1^; ^3^F_2_→ ^3^D_1_)
		687.8	Sr (5s^1^6s^1^ → 5s^1^5p^1^; ^3^S_1_ → ^3^P_0_)
barium	blue	455.4	Ba^+^ (6p^1^ → 6s^1^; ^2^P_3/2_ → ^2^S)
		493.4	Ba^+^ (6p^1^ → 6s^1^; ^2^P_1/2_ → ^2^S)
copper	green	510.6	Cu (3d^10^4p^1^ → 3d^9^4s^2^; ^2^P_3/2_ → ^2^D_5/2_)
		515.3	Cu (3d^10^4d^1^ → 3d^10^4p^1^; ^2^D_3/2_ → ^2^P_1/2_)
		521.8	Cu (3d^10^4d^1^→ 3d^10^4p^1^; ^2^D_5/2_ → ^2^P_3/2_)
		522.0	Cu (3d^10^4d^1^ → 3d^10^4p^1^; ^2^D_3/2_ → ^2^P_3/2_)

The emissions from alkali metal flames are generally composed of one or two prominent lines, whereas those of alkaline earth metals and copper consist of several lines. Table 1 lists only the most prominent of several lines for these systems.^26^ Transitions are indicated with electron configurations and also spectroscopic term symbols for more advanced readers. A more complete listing of the transitions for each metal species can be found in the NIST Atomic Physics site.^26^ This site also indicates the intensities of atomic transitions given as the corresponding Einstein *A* coefficient (the radiative rate coefficient) in units of s^–1^. Usage of the NIST site requires clarification of how physicists indicate atomic charge states, which is unfortunately not the same notation used by chemists. For example, a neutral sodium atom (Na) is indicated in the NIST site as Na I. A singly charged sodium ion (i.e., Na^+^) is indicated as Na II. A doubly charged barium ion (Ba^2+^) is indicated in the NIST site as Ba III. For every metal the neutral state is indicated as I, the singly charged state is II, the doubly charged state is III, etc. The NIST site gives the electron configuration involved in each atomic transition, eliminating any uncertainty. The NIST site also gives the energy levels of many atomic states whose transitions to the ground state are not allowed by optical selection rules, but a complete investigation of this spectroscopy is beyond the scope of the present discussion. Other compilations of atomic transitions and line intensities are also available.^32^

As indicated in Table 1, the emission transitions for most of these metals are those corresponding to the *neutral metal atoms* and not to the ions present in solution. This makes sense because the solutions containing the solvated ions are usually clear and colorless. Solvated ions such as Na^+^, Ca^2+^, etc. have closed-shell electronic configurations like those of the nearest rare gas atoms and therefore have transitions only at extremely high energies. Selected examples of allowed transitions for metal cations are given in Table 2, which all occur in the vacuum ultraviolet region of the spectrum (wavelengths shorter than 200 nm where the oxygen in air begins to absorb). Excitation of such high energy states is not efficient under flame conditions and even if those emissions did occur, they would not be visible to human eyes. In the case of barium, the emission is neither from the neutral metal atom, nor from the Ba^2+^ which exists in solution; it is from the singly charged Ba^+^ ion. Like the alkali metals, the emitting species has a charge reduced by one unit from that of the species in solution. Singly charged ions for the alkaline earth metals have a single unpaired electron in their valence shell like the neutral alkali metal atoms, and consequently have lower energy excited electronic states and transitions than the corresponding doubly charged ions. Neutral group II atoms may also emit, as in the case of calcium. The emitting species in these cases depends on subtle details of transition strengths and flame chemistry.

**Table 2:** Selected Atomic Emission Transitions for Representative Metal Cations

Metal	λ (nm)	Assignment
Li^+^	19.9	1s^1^2p^1^ → 1s^2^ (^1^P → ^1^S)
Na^+^	37.2	2s^2^2p^5^3s^1^ → 2s^2^2p^6^ (^1^P → ^1^S)
K^+^	60.1	3s^2^3p^5^4s^1^ → 3s^2^3p^6^ (^1^P → ^1^S)
Ca^2+^	40.4	3s^2^3p^5^4s^1^ → 3s^2^3p^6^ (^1^P → ^1^S)
Ba^2+^	65.7	5s^2^5p^5^6s^1^ → 5s^2^5p^6^ (^1^P → ^1^S)
Cu^+^	135.9	3d^9^4p^1^ → 3d^10^ (^1^P → ^1^S)

The unpaired electrons in the valence shells of neutral alkali atoms and singly charged alkaline earth ions produce spectra at low energies in the visible and/or near-UV wavelength regions. Additionally, under certain conditions, these unpaired electrons give rise to “spin-orbit interaction,” which causes atomic transitions to exhibit so-called “multiplet” splittings (see ref 33 for more details about this). Such splittings are observed for several of the transitions indicated in Table 1 in which the starting and ending electron configurations are the same, but the term symbols have different subscripts. This is the case for sodium, for example, which has two transitions associated with emission from the 3p state to the 3s level, indicated as ^2^P_3/2_ → ^2^S and ^2^P_1/2_ → ^2^S (see Table of Contents graphic). The theory of spin-orbit splitting is beyond the scope of General Chemistry, but these characteristic splitting patterns also provide unequivocal confirmation that the transitions are identified correctly.

Of the transitions mentioned in Table 1, that for sodium is particularly noteworthy. The closely spaced spin–orbit doublet of neutral sodium atoms near 589 nm has a long history in atomic spectroscopy. This is the famous sodium “D” line first observed by Fraunhofer in his spectrum of the sun.^2,34^ This wavelength is in the middle of the optical spectrum and has become a standard wavelength for optics, used to tabulate refractive indices of chemicals or materials, focal lengths of lenses, and other optical parameters which vary with wavelength. For example, refractive indices of chemicals vary with wavelength and so the index value at 589 nm, indicated as η_D_, is tabulated in databases. This sodium “D” line is also seen in the spectra of almost all stars and its Doppler shift is used to determine the velocity of those stars relative to earth.^28^

Having established that the emitting species in flame tests are not the ions which exist in solution, it is only natural to ask how it is that lower charge states are produced and excited into the states which emit. We use NaCl solution as the example. When this salt is dissolved in water, the aqueous Na^+^(aq) and Cl^–^(aq) ions are produced in solution. This solvation process is already familiar to students in General Chemistry, although it is well documented that many students do not understand its details.^35–38^ The reverse of this process happens in a flame test. The heat of the flame removes the water molecules of solvation, and then the naked Na^+^ and Cl^–^ ions in the gas phase are relatively unstable. Recombination of these ions to produce the NaCl diatomic molecule is possible. However, if this does occur, the excited states lie at high energy and are not excited efficiently. If such states are excited, the emission spectrum of NaCl is in the ultraviolet region and not visible to human eyes.^39^ However, collisions between the Na^+^ and Cl^–^ ions can also result in the process of electron transfer. The attractive binding interaction for a neutral atom with its outermost electron is the ionization energy, and that for an anion is the electron affinity. The ionization energy of a sodium atom (5.14 eV; 495.5 kJ/mol) is greater than the electron affinity of a chlorine atom (3.62 eV; 349.3 kJ/mol) and so the extra electron of Cl^–^ transfers back to the Na^+^ ion, neutralizing it. The exothermicity of this reaction, together with the thermal energy in the flame, produces the neutral Na atom in its excited electronic state, which then emits. The chlorine atom has no low energy excited states and therefore does not produce any visible emission. This suggested mechanism is summarized in Figure 1. Like most other mechanisms in chemistry, these details are difficult to prove conclusively but are plausible and consistent with the known observations. The specific details of this kind of desolvation and electron transfer are different for every metal salt solution because of different ionization potentials and electron affinities of the elements and the different energies of the metal excited states, but the qualitative idea is the same.

**Figure 1:**
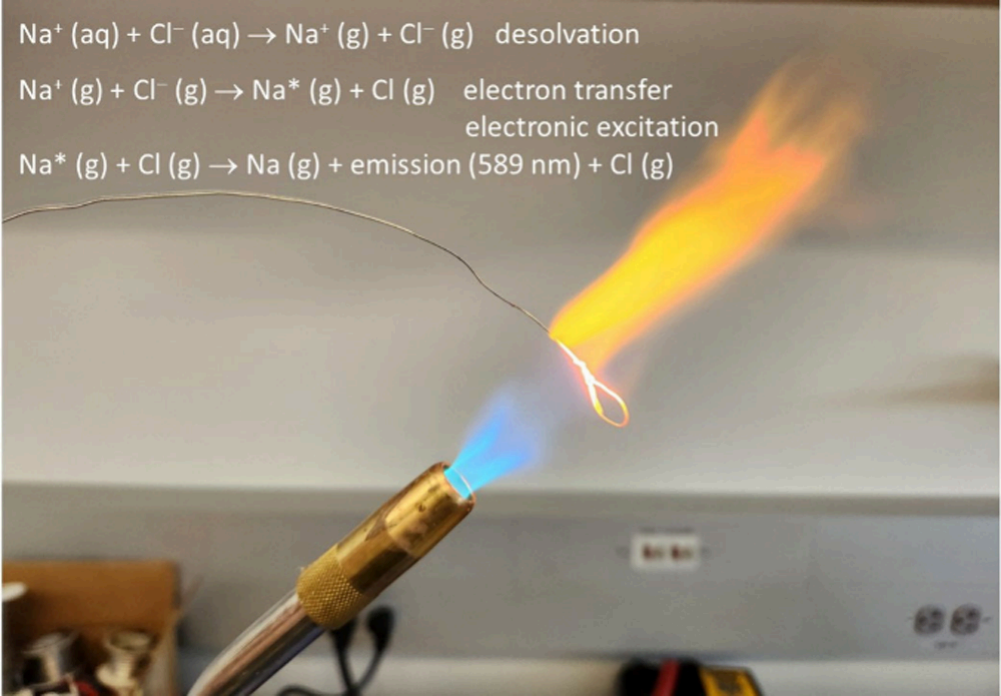
Flame emission resulting from a NaCl solution. The yellow emission is from neutral sodium atoms via the so-called “D” line at 589 nm.^26^

In conclusion, well established atomic spectroscopy shows that the emitting species in common flame tests are not the ions which exist in solution. Instead, the emitting species are usually the corresponding neutral metal atoms, and occasionally the corresponding metal ions with a reduced charge from that in solution. The excited states of these metal species are produced via desolvation and reverse electron transfer from the anions in solution to the cations, neutralizing them or reducing their charge, which results in the excited states which emit.

How then, can these ideas about flame tests be taught more effectively? Several concepts seem to be critical for this. First of all, it is important to emphasize to students that neutral atoms and their ions are very different chemical species—the gain or loss of an electron is a spectacular event for an atom with a tremendous impact on its energy states and spectra. Positive ions attract electrons much more strongly than neutral atoms. This stronger interaction means that moving electrons to excited orbitals for ions requires much greater energies, often beyond those of visible light. Second, the color of objects in nature, such as salt solutions, is directly related to the energy levels of their component species. Salt water is clear and colorless because the ions involved have no low energy states corresponding to visible light absorption or emission. It is also important to introduce students to the NIST Atomic Physics Web site early in their careers. This allows them to check atomic transitions and energy levels on their own. Finally, more emphasis on atomic spectroscopy and its connection to chemical analysis, lasers and astronomy may motivate students to further pursue these topics in advanced chemistry classes.
